# Microorganisms harbor keys to a circular bioeconomy making them useful tools in fighting plastic pollution and rising CO_2_ levels

**DOI:** 10.1007/s00792-022-01261-4

**Published:** 2022-02-03

**Authors:** Garabed Antranikian, Wolfgang R. Streit

**Affiliations:** 1 Center for Biobased Solutions (CBBS), Hamburg University of Technology, Hamburg, Germany; 2 Department of Microbiology and Biotechnology, University of Hamburg, Hamburg, Germany

**Keywords:** Biotechnology, Biocatalysis, Biotransformations, Industrial applications, Circular bioeconomy, Microbial plastic removal

## Abstract

The major global and man-made challenges of our time are the fossil fuel-driven climate change a global plastic pollution and rapidly emerging plant, human and animal infections. To meet the necessary global changes, a dramatic transformation must take place in science and society. This transformation will involve very intense and forward oriented industrial and basic research strongly focusing on (bio)technology and industrial bioprocesses developments towards engineering a zero-carbon sustainable bioeconomy. Within this transition microorganisms—and especially extremophiles—will play a significant and global role as technology drivers. They harbor the keys and blueprints to a sustainable biotechnology in their genomes. Within this article, we outline urgent and important areas of microbial research and technology advancements and that will ultimately make major contributions during the transition from a linear towards a circular bioeconomy.

## Introduction

The major global and man-made challenges of our time are the fossil fuel-driven climate change (Lelieveld et al. 2019, Karl and Trenberth 2003; Cavicchioli et al. 2019), a global plastic pollution (Haward 2018) and rapidly emerging plant, human and animal infections (Baker-Austin et al. 2017; Anderson et al. 2020; Blum and Hotez 2018).

**Fig. 1:**
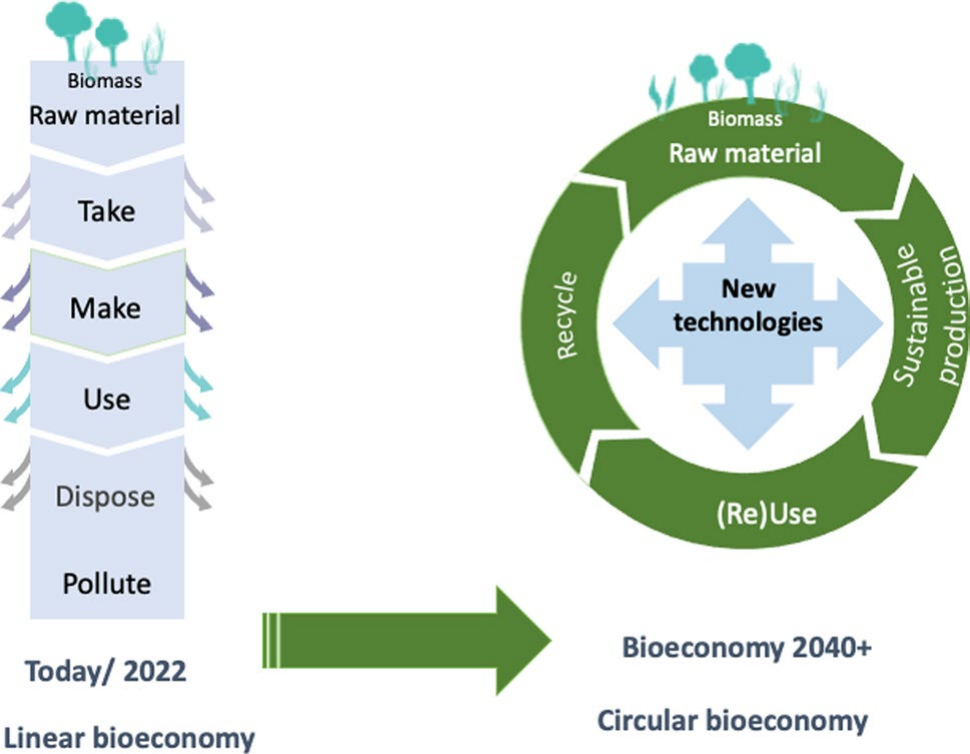
Linear versus circular bioeconomy

One solution that addresses many of these challenges is to master a rapid transition within 20–30 years from a linear economy to a sustainable, biobased circular economy (Fig. 1). To meet the necessary global changes, a dramatic transformation must take place. This transformation will involve very intense and forward oriented industrial and basic research strongly focusing on (bio)technology and industrial bioprocesses developments.

Within this transition microorganisms—and especially extremophiles—will play a significant and global role as technology drivers. They harbor the keys and blueprints to a sustainable biotechnology in their genomes. Any technology advancement will need to make use of the billions of microbial (bio)catalysts, pathways, microbial cells, consortia, compounds, and artificial cell fabrics. Traditionally, microorganisms have delivered a wide diversity of enzymes (Bell et al. 2021; Elleuche et al. 2014; Littlechild 2015) and are responsible for 70% of the globally used antibiotics (Kumar et al. 2017; Fischbach and Walsh 2009). Now it can be expected that their role will go far beyond this. Part of this is founded by their enormous diversity. It is well known that over 10^30^ microorganism live on this planet of which the majority has not yet been cultivated (Whitman et al. 1998; Lloyd et al. 2018). Both, the cultivated and non-cultivated microorganisms harbor an unlimited number of useful biocatalysts and secondary metabolite pathways (Ferrer et al. 2016; Streit and Schmitz 2004). This nearly unlimited natural diversity combined with artificial evolution and engineering technologies (Packer and Liu 2015; Arnold 2018) is the backbone of modern biotechnology and future bioindustries. It allows the steady development of more advanced and truly sustainable industrial processes and valuable biomolecules. To achieve this, a high level of innovations is indispensable.

**Fig. 2:**
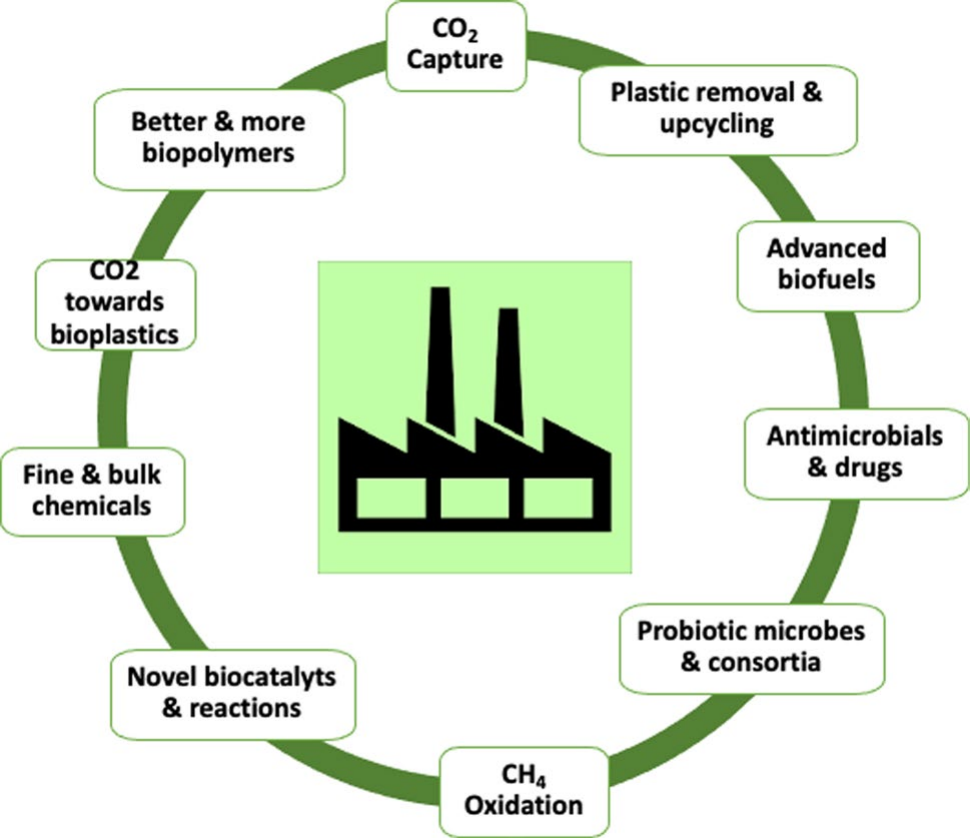
Key technology developments needed for a sustainable economy and that are driven by microorganisms. Only the most pressing challenges are depicted. Useful references are given in Table 1

Below and in Fig. 2, we outline some urgent and truly important areas of research and technology advancements that need to be addressed and with respect to the most pressing challenges society faces. In these fields microbial biotechnology will ultimately make major contributions to the societal and industrial transition:

**Table 1:** Some top challenges to which microbial enzymes can contribute towards a sustainable bioeconomy in the next decade

Biological process	Technical challenges/solutions and innovations needed	Highly accessed reviews and spotlight paper in the field
Decarbonization: capturing CO_2_	Developing efficient in vivo and in vitro processes using microbial CO_2_ fixing enzymes	Appel et al. (2013), Lemaire et al. (2020), Erb and Zarzycki (2016)
Decarbonization: oxidizing CH_4_	Developing efficient in vivo and in vitro processes using methane oxidizing enzymes	Sirajuddin and Rosenzweig (2015), Lawton and Rosenzweig (2016), Clomburg et al. (2017)
Plastics removal from the environment and establishing recycling concepts for circular use	Identifying enzymes acting on polymers, such as PE, PVC, PS, PA and PU; upscaling and optimizing processes for PET and ester-based PU but also epoxy-based polymers	Danso et al. (2019), Wei and Zimmermann (2017), Urbanek et al. (2018)
Generating high quality and large amounts of versatile bioplastics	Reducing costs for production, increasing production scale; defining better natural polymers with better properties in designer bugs; optimize harvesting from cells	Di Bartolo et al. (2021), Choe et al. (2021), Zhao et al. (2020), Raza et al. (2018)
Providing truly renewable liquid and gaseous fuels from 2nd and 3rd generation	Hydrolysis of complex lignolytic plant material, better performing (engineered) microbial communities or single organisms. Obtain better genetic tools to manipulate and engineer alga and to improve 3rd generation biofuel production; improved tools to engineer bacteria/archaea involved in biogas and bioalcohol production	Lynd (2017), Callegari et al. (2020), Shuba & Kifle (2018), Milano et al. (2016), Keasling et al. (2021)
Providing sustainable nontoxic biocides or probiotic microbes for plant and animal protection	Establishing efficient synthesis routes using novel enzymatic activities; solvent tolerance Identifying probiotic communities or single organisms	Yi et al. (2021a, b), Lee et al. (2012), Bell et al. (2021)
Identifying novel biocatalytic reactions for synthesis of fine and bulk chemicals	Identify more and better enzymes forming amide and C–C bonds; enzymes acting on ether bonds; increase available transferases, halogenases and others Better host strains, explore the dark matter proteins with no assigned function	Rosano and Ceccarelli (2014), Hauer (2020), Intasian et al. (2021)
Digitalization and tool development for faster engineering, enzyme delivery advancing ultrahigh throughput enzyme mining approaches	Investing in and developing enzyme technology driven platforms, developing machine learning and automatization	Damborsky and Brezovsky (2014), Qu et al. (2019) Mazurenko et al. (2020), Dörr et al. (2016), Markel et al. (2020), Colin et al. (2015)

## The future challenges

### Combining biodiversity with synthetic biotechnology for CO_2_ capture at industrial scale

The fossil-energy driven CO_2_ emission and the resulting increased global warming are one of the most important challenges we currently face (Karl and Trenberth 2003; IPC 2014). It will affect life of the next generations at unprecedented levels. The increasing number of droughts, wildfires, heat waves in southern countries, heavy rain fall, flooding are first signs of a human-made climate change.

Thus, it is now the time to start looking for solutions to this challenge. Therefore, developing bio-based CO_2_ capture technology at industrial scale will be a very urgent task. Notably, several different chemical or electrochemical technologies have been developed over the years (Qiao et al. 2014). While these are often highly effective, they require input of high amounts of energy and recycling of metal catalysts. These requirements make them not as sustainable as needed.

Notably, the blueprints for sustainable CO_2_ capture processes are encoded in the microbial genomes. Currently we know 7 different pathways involved in CO_2_ fixation and we can exploit them for bioprocesses linked to decarbonization. Perhaps the best-known pathway is the one used by the green plants, algae, the cyanobacteria, and related microorganisms. At the core of this process the enzyme ribulose-1,5-bisphosphate carboxylase/oxygenase (RubisCO) is found as part of the Calvin–Benson–Bassham cycle (Erb and Zarzycki 2018). In addition, one of the evolutionary very early pathways is the Wood–Ljungdahl pathway. It is well conserved within the acetogenic bacteria and the methanogenic archaea and is often associated with extreme habitats (Ragsdale and Pierce 2008; Schuchmann and Müller 2014; Drake et al. 2008). Furthermore, nature has evolved few other highly efficient pathways to fix CO_2_ from the atmosphere among them the 3-hydroxypropionate bicycle, the 3-hydroxypropionate/4-hydroxybutyrate cycle, dicarboxylate/4-hydroxybutyrate cycle (DC/HB cycle) and the reverse tricarbonyl acid cycle (Fuchs 2011; Berg 2011; Nunoura et al. 2018; Song et al. 2020; Mall et al. 2018; Steffens et al. 2021). Altogether these pathways and their enzymes will be important study objects for the design of artificial CO_2_ capture systems. However, relatively low number of pathways identified and related to CO_2_ capture may indicate that we have not yet fully uncovered the diversity of pathways that can contribute to this process. Thus, we should set out to identify the full repertoire of nature’s toolbox of CO_2_ but also CO fixing enzymes and pathways. Further understanding the molecular and structural mechanisms will advance our approaches with respect to the design of synthetic organisms and processes needed for industrial CO_2_ capture.

Thereby studying biochemical and structural aspect of all key enzymes involved in CO_2_ fixation is essential.

One of the key enzymes involved in CO_2_ fixation and which is present in all living organism is the carbonic anhydrase (CA) (Alvizo et al. 2014). CA is a remarkable enzyme. It is one of nature’s fastest enzymes as it can turnover CO_2_ and water to bicarbonate and a proton at a rate of almost a million reactions per second (Zhang et al. 2013; Blais and Rogers 2003). Thus, it is an ideal candidate for CO_2_ capture processes. This has for instance been shown in combining the CA with a bioelectrocatalytic process and an oxidoreductase to produce methanol using CO_2_ (Addo et al. 2011).

First attempts towards the development of highly effective and CO_2_-fixing and synthetic microorganisms have been made and are promising. They are reviewed in (Erb and Zarzycki 2016; Gong et al. 2016; Bar-Even et al. 2010; Scheffen et al. 2021; François et al. 2020; Appel et al. 2013). Notably, these pathways are not yet economically feasible due to various reasons (Claassens 2017) but as this is an emerging field of research it can be expected that it will rapidly advance and solve some of the problems.

Thereby using CO_2_ to produce truly degradable biopolymers might be solution that solves two problems. It reduces the global CO_2_ emission and makes significant contribution to the advances towards plastics removal (Lemaire et al. 2020).

Artificial CO_2_ capturing systems can, however, only have an impact if they are set up at truly large scale and capturing at least 0.5–1 GT of CO_2_ per year. To establish such large-scale processes at industrial scale they need to be economically feasible and generate jobs at different levels. Within this setting, the introduction of a price tag for CO_2_ within the European Union is one step into the right direction. A ton of CO_2_ currently costs approximately 30 € within the European Union, and it is estimated to have a market price of 120 € per ton to achieve the major decarbonization goals. Putting a price on CO_2_ will ultimately result in the generation of a global market. Thus, developing sustainable processes that allow industrial CO_2_ fixation and either incorporation in renewable materials or long-term storage should not only be financially quite rewarding but also rewarding with respect to achieving major decarbonization goals.

Consequently, developing biobased decarbonization systems at truly large scale up to several hundred million tons per year could be a first primary goal.

### Methane removal from the atmosphere

Methane is a very strong climate gas. It has a 25-fold higher impact on the global warming compared to CO_2_ as it absorbs higher levels of solar radiation (Le Mer and Roger 2001). Next to CO_2_ methane is the second most abundant greenhouse gas and it accounts for a minimum of 25% of current global warming. However, it is not as resilient as CO_2_ and remains only for about 10 years in the atmosphere.

It is released at relatively high levels from perm frost soils but also from several other sources including insect guts, biogas plants, ruminants, leaks in pipelines, rice paddies (Cusworth et al. 2021; Pétron et al. 2012; Le Mer and Roger 2001). Interestingly, landfill appears to be responsible for 22% of the overall methane emission in industrializes countries (Scheutz et al. 2009; Sadasivam and Reddy 2014) and oil and gas industries appear to be main producers as well (Ocko et al. 2021).

Currently only a small part of methane is used as energy carrier. In addition, methane is the primary source for H_2_ (Chen et al. 2020). Thus, making better and more sustainable use of methane from biogas plants, industries or landfills other than energetically is an intriguing challenge. Thereby the production of biofuels or higher value chemical compounds and building blocks should be one primary goal (Haynes and Gonzalez 2014).

Several chemical processes have been established that make use of CH_4_. However, the activation of the C–H bonds by chemical processes is a challenge and requires relatively high levels of energy (Foster 1985; Enger et al. 2008). In the contrary microorganisms have found ways to handle methane at relatively mild temperatures and low pressures. Thereby the oxidation of methane to methanol is perhaps the fastest route to solve the problem (Haynes and Gonzalez 2014).

CH_4_ is oxidized under aerobic but also under anaerobic conditions (Hakemian and Rosenzweig 2007). The key enzyme involved is the methane monooxygenase. It is a heterotrimeric enzyme and present in a wide range of phylogenetically diverse microorganisms. The primary product of the oxidation is methanol (Banerjee et al. 2019). Methanol can be used in fuel cells but also to produce higher value chemical compounds either using a metal catalyst and/or using an enzymatical process.

Establishing any process linked to large scale production of methanol or chemical compounds based on methane faces several major challenges. First the gas is only poorly soluble in water and thus the diffusion rates are a major limiting factor during the process. Second and the perhaps more critical problem is linked to the fact that methane is a gas that burns in the presence of oxygen and if not handled carefully explosions can occur. Furthermore, methanol is toxic at higher concentrations. Therefore, enzymes and or microbes involved in any large-scale technical process need to be tolerant to it up to a certain level. These are some of main technical challenges that currently hinder the development of larger scale bioprocesses using methane.

Therefore, developing technologies that reduce the methane load within the atmosphere is a pressing but also demanding task. Within this framework construction smart biofilter systems affiliated with landfills to oxidize the methane to methanol are truly rewarding and helpful projects (Scheutz et al. 2009).

One forward approach should include the introduction of a tax on methane in a similar way as it has been established for CO_2_. This would ultimately allow the development of a global market and advance any processes towards a biobased methane removal.

### Plastic removal and degradation in marine and terrestrial environments

Petroleum based plastics are designed to be extremely stable and durable. Currently about 360–450 million tons of synthetic polymers are produced and much of this is only meant for single use (Plasticseurope 2018). The global market is expected to be around 500–600 Billion € (MacArthur Foundation).

Recycling concepts hardly exits and more than 90% of all synthetic and fossil-derived plastics end up in either landfills or directly in the environment. The over 70 year-long global use at a multi-million tons scale and the lack of global concepts for recycling and circular use has resulted in unprecedented pollution in nearly all environments. Plastic litter will affect our well-being and the biodiversity at various levels (Jambeck et al. 2015; Gall and Thompson 2015; Kühn et al. 2015; Wilcox et al. 2015). The MacArthur foundation has estimated that already in 2050 more plastic particles will be present in the ocean than fish (Foundation 2017).

To face this dramatic challenge, we need to rethink the design and uses of plastics, and it is mandatory to establish new concepts to facilitate better microbial degradation and circular use. However, the knowledge on microbial plastics degradation is rather sparse. Microbial and enzyme driven plastics degradation has only been studied since less than a decade. Several excellent reviews have summarized the current knowledge on enzymatic breakdown (Danso et al. 2019; Wei and Zimmermann 2017; Urbanek et al. 2018). Based on these the main plastic litter we face is composed of either single or mixed materials consisting of the polymers: Polyurethane (PUR), Polyethylene (PE), Polyamide (PA), Polyethylenterephthalate (PET), Polystyrole (PS), Polyvinylchloride (PVC), Epoxy-based polymers (EP) Polypropylene (PP) and tire rubber.

With respect to a possible microbial degradation, we know few enzymes and microorganisms acting on PA oligomers, ester-based PUR and PET. Today, however, no verified enzymes are known that degrade the polymers PE; PVC; PP; PS, EP and ether-based PUR (Danso et al. 2019). These polymers stand for the majority of all synthetic polymers. Thus, the identification of microbial enzymes and pathways acting on most of the current polymers is a major and urgent task.

In addition, developing biotechnological processes that degrade mixed plastics at industrial scale is certainly a further challenge. In the meantime, finding and deciphering major pathways involved in the degradation of micro- and nanoplastics in the environment will also be a highly rewarding contribution.

PET degradation is currently the best studied model polymer with more than 30 enzymes known (Zhang 2022). It should be kept in mind that even for this model system we still have huge knowledge gaps with respect to the molecular details of enzymatic PET degradation. We basically do not know how the enzymes bind to the fibers and how the enzymes affect the crystallinity of the polymer. It is also not well understood if these enzymes are endo- or exo-cleaving. Notably in nature it cannot be expected that a polymer degradative pathway is induced. It is rather that we would expect that highly promiscuous enzymes by chance attack the fibers and release small amounts of the monomers. These are only few questions that need to be addressed in future research.

Therefore, the identification of novel plastic-active microorganism and enzymes is an urgent task. Once we fully understand the molecular and structural mechanism of polymer hydrolysis, we can make use of it in industrial processes at large scale. In fact, we can advance the synthesis of these polymers using this knowledge to make them better biodegradable.

Recently, it has been shown that PET can be efficiently converted to vanillin (Sadler and Wallace 2021). Thereby, making use of this waste and producing higher value chemicals is certainly a worthwhile concept. Consequently, future concepts need to be developed to make better circular use of plastic waste. This should include the design of engineered strains that are able to deliver a wide range of chemical compounds when fed on synthetic polymers.

### Designing degradable and durable industry usable biopolymers

As mentioned above we face a global plastic crisis. The main challenge is that we rely on plastics for our daily life and our economy. However, simply saying, we don’t want to use them any longer is not a perspective. One smart way out of this dilemma is to develop bio-based strategies for the synthesis of truly biodegradable polymers. Today the best known examples of so-called biodegradable polymers are, poly-beta-hydroxybutyrate (PHB), poly(lactic acid) (PLA), poly(glycolic acid) (PGA), poly(lactic-co-glycolic acid) (PLGA), poly(butylene succinate) (PBA), polycaprolactone (PCL), poly(ethylene adipate) (PEA), poly(p-dioxanone) (PDS). These polymers have already been implanted in the circular bioeconomy concepts and are used at increasing levels (Morell 2019). Their main field of applications, however, lies in either the medical area for drug delivery, wound coverage, and or composting bags and packaging material. Notably, none of these polymers has made it into high end applications (Di Bartolo et al. 2021; Choe et al. 2021; Zhao et al. 2020; Raza et al. 2018).

Thereby today’s bioplastics production equals less than 1% of the overall synthetic polymers produced (https://www.european-bioplastics.org/market/). Thus, increasing their production and use by reducing the use of the fuel-based polymers is´ a main challenge.

Besides these the chemical or enzymatic modification of so-called natural polymers such as chitin, cellulose, alginate, glycogen, PHBs, PHAs, DNA, proteins and/or starch result in the production of semi-synthetic polymers with highly sophisticated biodegradative capabilities. The design of these is as well an attractive target.

To further make advancement in this field we need additional biopolymers with better traits with respect to durability, elasticity, and longevity. They must compete in their material and physical properties with the currently used ones that are made from fossil fuel.

One way to achieve this ambitious goal is to modify current polymers by introducing breaking points and by implanting stable enzymes into the fibers that can be activated after some time to initiate the degradation. Thus, combining enzyme-mediated with chemo-enzymatic synthesis is a very realistic long-term goal (Panchal & Vasava 2020; Haider et al. 2019).

In theory it should also be possible to teach an organism to synthesize a biodegradable PET by reversing the reaction of the known PET hydrolases. Similar strategies could be applied for other polymers. Especially enzymes from extremophiles could be the key to the synthesis of such polymers as they are expected to work under the almost water free conditions in organic solvents.

### Renewable biofuels, energy carriers H2, ethanol, methanol

Today the transport sector is responsible for 15% of the global CO_2_ emission (Sims et al. 2014). This sector relies heavily on fossil-based fuels with more than 96% of all fuels originating from fossil resources (IEA 2019).

Since the 1940s much interest has been paid to the development of different types of biofuels. Thereby the two main types of liquid biofuels that are produced and sold are biodiesel and bioethanol. Furthermore, short chain alcohols and other compounds are listed as biofuels. Among these are butanol and methanol perhaps the most important ones. In addition to these liquid fuels, hydrogen and methane are considered as important gaseous biofuels.

Biofuels are classified according to their origin and production. The biofuels produced from crops, food and animal-feed are referred to as first-generation biofuels. Second generation biofuels are derived from so called energy crops, such as *Miscanthus* (switchgrass) but also other non-food plants, agricultural waste and other lignocellulose biomaterial containing waste often originating from forestry.

These 1st and 2nd degree biofuels are in principle generated by microbes and relying on their hydrolytic activities, fermentation, but also large-scale transesterification (Oh et al. 2018).

Furthermore, technical processes are implanted at different levels including in part gasification, pyrolysis, distillation, Fisher–Tropf process and others. Notably the further improvement of the main microbial and enzymatic steps involved in combination with chemical and physical steps is certainly a worthwhile task. One of main bottlenecks is still linked to the hydrolysis of the plant and waste material (Lynd 2017; Callegari et al. 2020; Shuba and Kifle 2018; Milano et al. 2016).

Biofuels of the third generation are produced directly by microalgae. They synthesize biodiesel as part of their metabolism. In addition, transesterification is employed (Keasling et al. 2021). Thus, identifying best suited microalgae, making them genetically accessible and further improving their biodiesel production rates are the most urgent goals.

The advantage of the microalgae is linked to their simple growth requirements. They can be fed with wastewater and can virtually be grown in all environments. In fact, they do not compete with the food and feed industries for resources and could be grown on landfill areas, that can hardly be used for anything else. The main bottleneck, however, is the genetic accessibility to engineer better performing algae. Thus, the development of additional process involved in the sustainable biofuels production is a major challenge.

Thereby, it should be kept in mind that the overall role of biofuels has been critically discussed in the past because of their overall life cycle assessments. These have delivered controversial results with respect to their overall impact on climate change and CO_2_ burden and this should be taken into account in any future attempts (Jeswani et al. 2020).

### Bioelectrodes from bacteria

Bioelectrochemically active bacteria are well known for their capability to generate small amounts of electricity. These systems have become quite attractive in recent years, because they offer the fascinating possibility to generate electric power by simply fermenting sugars and/or cheap waste (Zheng et al. 2020; Sydow et al. 2014). The overall concept is to engineer bioelectrochemical systems (BES) for either the use as microbial fuel cells (MFCs) and/ or for the microbial electrosynthesis (MES). Using these systems for either electrogenesis and/or chemical synthesis is a future challenge (Xie et al. 2015; Hirose et al. 2018).

The best studied model systems are *Geobacter* and *Shewanella*. Both organism can discharge respiratory electrons to insoluble Fe(III) or Mn(IV) oxides. They have evolved mechanisms to transport electrons from inside the cell to extracellular electron acceptors. For this they can use flavins, nanowires and outer membrane c-type cytochromes (Shi et al. 2016; Kumar et al. 2017; Dundas et al. 2018). While it can be assumed that many microorganisms are capable to perform this exogeneous electron discharging, the main challenge is linked to upgrading the diverse systems to industrial scale.

### Production of novel pharmaceutical compounds, cosmetics, antibiotics, drugs biocides and chiral compounds

#### Enzymes as global biocatalyst to address biosynthesis and hydrolysis and modification

Traditionally microorganisms have been a vast source for enzymes and secondary metabolites. Enzymes as biocatalysts play an important role for the production or modification of food and animal feed, textiles, detergents, fine and bulk chemicals in pharmaceuticals, cosmetics, and other industries. Other fields include paper and pulp and leather processing but also agriculture. The estimated global market will be reaching a value of 14,507 billion USDs in 2027 from 8636 billion USDs in 2019 with an annual growth of 6.5% (https://www.alliedmarketresearch.com/enzymes-market).

Alone for agriculture the global enzyme market has been estimated to be $616.5 Million by 2026 from 346 in 2020 (https://www.strategyr.com/market-report-agricultural-enzymes-forecasts-global-industry-analysts-inc.asp).

Today we have a large toolbox of enzymes from all seven enzyme classes (EC 1–7). Notably, the lipases/esterases and ketoreductases have been the preferred enzymes in modern bioprocesses to produce, separate or modify fine chemicals. Carbohydrate active enzymes (also called CAZymes or carboactive enzymes, carbozymes often glycosyl hydrolases) such as cellulases, xylanases and amylases are the working horses in the washing powder and food industries together with proteases and the demand for novel enzymes with better or different features is still very high.

Besides these polymerases, DNA and RNA binding proteins and nucleases will play an increasing role especially for the detection of novel viruses and emerging diseases. The nucleases will be of extreme value for precision targeting of genomes as recently demonstrated for CRISPR Cas. Recently and because of the high demand of COVID 19 vaccines RNA polymerases will be of importance because of their role in mRNA vaccine production. Further nucleoside sugar transferases, sulfatases, kinases and other carbohydrate or polyphenolic compound modifying enzymes will be of increased value for future biotechnological applications. In addition, enzymes modifying synthetic or natural compounds, such as glycosyltransferases, will play an important role in the global and sustainable bioindustries.

For some type of reactions, we either lack or simply have not enough enzyme diversity. Among those are halogenases, C–C ligases, transaminases, epoxy-active enzymes, ether-bond active enzymes but also enzymes acting on C=N bonds and others (Hauer 2020).

In general enzyme promiscuity may be a key to obtain better performing enzymes and allrounders and to have a quicker access into the market (Martínez-Martínez et al. 2018; Khersonsky & Tawfik 2010).

While often the WT enzymes have not the desired catalytic activities or selectivities, the design of tailor-made enzymes in combination with smart mining approaches has become a very important and truly successful tool to deliver novel catalysts.

Direct evolution approaches have helped us to quicky adopt and alter enzyme activities (Bell et al. 2021). Thus, the development of additional structure related technology to optimize enzymes activities will be of great value and further multiply the already existing natural biodiversity by several factors.

Notably, half of the proteins in non-cultivated organism have no function predicted. They are often referred to as dark matter proteins. While few researchers would argue that we cannot make use of these not-characterized proteins, one could, however, argue that these proteins represent a large pool of sequence space with novel catalytic capabilities that need to be exploited. Therefore, the exploitation of this pool of dark matter proteins will advance the field of biotechnology.

Since industry often needs catalyst that function in the presence of higher temperatures in the presence of solvents or increased salt conditions, elevated pressure, or at very low temperatures it can be expected that especially microorganisms adapted to more extreme conditions will deliver very useful and versatile biocatalysts and solutions to one or several of the major global challenges we currently face. This lies in part in the higher stability of enzymes but is also a direct cause of their excellent adaption to extreme conditions.

#### Secondary metabolites from bacteria built the backbone of pharmaceutical products

Next to enzymes, secondary metabolites from microorganisms are a major field of research.

The world production and detection of antibiotics heavily relies on bacteria. The Gram-positive *Streptomyces* and closely related species still deliver > 70% of all antibiotics (Kumar et al. 2017; Fischbach and Walsh 2009). In fact, the current antibiotics have been delivered by a rather small group of microorganisms. However, the non-cultivated majority offers a by far larger resource (Crits-Christoph et al. 2018; Kautsar et al. 2020).

In view of quickly emerging pathogens and since only few novel antibiotics are currently in clinical test trials, the exploitation of this biological source is certainly a very important future task. The use of this secondary metabolite reservoir will, however, go beyond the clinical applications. Many of these compounds could be the blueprints for true ‘biocides’ with less impact on the environment used in industry or agriculture.

In addition, it should be kept in mind that besides enzymes and secondary metabolites, microorganisms produce a set of diverse compounds that are of importance to bioindustries. Among those are rhamnolipids, modified sugars, polyphenols often glycosylated and that are most wanted by industries. Exploiting the biosynthetic pathways further and making use of them for the next wave of biotechnology is an important and rewarding task.

### Novel cultivation approaches, identifying and applying probiotic and other valuable microorganisms

Traditional approaches have mainly used single organisms in microbiological research and production processes. However, developing stable microbial species communities (biofilms, consortia) to produce chemical compounds is certainly a future field of research. Clearly, many of the secondary metabolites and enzymes are only expressed in cocultures together with other microorganisms. To address these challenges cultivation approaches are needed that go beyond the classical single isolate ad strain concept.

Next to these the identification and cultivation of microorganism with strong probiotic traits will be an emerging field of research of future microbiologist. This arises from the need to manage and control microorganisms without applying chemical compounds. This is especially a challenge in agroindustry, where the use of biocides is due to health and environmental concerns strictly regulated. However, it will also be of importance in other fields such as the health-related areas controlling and managing the human microbiome with the goal to prevent pathogens and/or to change nutritional use of the diet. The same concept can be applied in animal health related issues.

Thereby, we will substitute the use of some antibiotics or biocides with beneficial microorganisms without the disadvantages of applying antibiotics or biocides to humans, animals, and plants.

Finally, the not yet cultured microbes as a major source for novel genes, pathways, and biocatalyst but also secondary metabolites will be a tremendously valuable resource. These need to be exploited. This will in part only be possible, if novel cultivation technologies are developed making use of automated high throughput cultivation and high level of digitalization.

### Uncultured microbes as a major source for novel genes and biocatalyst but also secondary metabolites: advanced biomining technologies

Over the last 20 years significant advances in the genomics of single bacteria and microbiota of humans, plants and animals have been made. These have tremendously increased our understanding on the diversity of microorganisms in general. Today, we know that the majority of these microorganisms remains uncultivated (Lloyd et al. 2018; Steen et al. 2019; Murray et al. 2020). Large scale genomes sequencing of metagenomes such as the Tara ocean project  have allowed us access to new sequences encoding completely new enzymes and reactions that will built the basis for future biotechnology and that need to be exploited (Daniel 2005; Nayfach et al. 2021).

Digital methods and big data handling have found their way into the life sciences, creating great potential in various research areas and especially in mastering the challenges described above. They will further make significant contribution to conserving natural resources and thus sustainably securing food and healthcare supplies as well as the provision of renewable energies for future generations.

Further the transition from single to multi-omics approaches will have a significant impact on the discovery of unique biological systems. Implementing high throughput mining approaches combined with in vitro technologies, directed evolution, deep mutational scanning, microfluidics and others will certainly further advance this field (Markel et al. 2020; Yi et al. 2021a, b).

### From biodiversity to the bioeconomy

The true potential of our planet’s biodiversity, especially of microorganisms growing under extreme conditions, for application in industrial biotechnology has so far been little exploited. Nevertheless, the above-mentioned approaches and strategies offer the potential to optimize the use of renewable raw materials in the bioeconomy and thus come a decisive step closer to the zero-waste goal.

A smooth transition can only be accomplished when paying attention to lessons learned from the past, such as avoiding future conflicts between food and fuel and avoid one-sided technology-driven approaches. In addition, lacking public acceptance of new technologies should be kept in mind when promoting a biobased industry. The initiation of new interdisciplinary network programs involving research centers, industries, politics and society have to be accomplished to meet the future challenges and contribute to the Sustainable Development Goals (SDGs) of the United Nations (https://sdgs.un.org/goals).

### Integrated approach and networks

The above-mentioned global challenges require concerted actions of different actors from politics, science, business, and civil society. An interdisciplinary approach involving different fields of natural and engineering sciences (such as microbiology, molecular biology, genetics, agronomy, chemistry, physics, process engineering) as well as logistics, digitalization and automation (robotics, artificial intelligence) increases the chances of realization and promises more robust solutions. Other factors such as knowledge transfer, entrepreneurship, communication, education, social skills, and sustainability are crucial for the transformation to bio-based technologies of the future.

To strengthen these urgently needed innovations, interdisciplinary research programs should be initiated and massively supported by governments, in close cooperation between the ministries of science and economics and universities. More such programs should also be launched and, therefore, be able to develop greater innovation potential.
